# Salvage Radiosurgery for Optic Nerve Sheath Meningioma

**DOI:** 10.7759/cureus.16450

**Published:** 2021-07-18

**Authors:** Kunal Vakharia, Hirotaka Hasegawa, Scott L Stafford, Michael J Link

**Affiliations:** 1 Department of Neurosurgery, University of South Florida, Tampa, USA; 2 Neurosurgery, Mayo Clinic, Rochester, USA; 3 Department of Neurosurgery, University of Tokyo, Tokyo, JPN; 4 Radiation Oncology, Mayo Clinic, Rochester, USA; 5 Neurological Surgery, Mayo Clinic, Rochester, USA

**Keywords:** salvage radiosurgery, optic nerve sheath meningioma, meningioma, stereotactic radiosurgery, gamma knife, optic nerve, radionecrosis

## Abstract

Optic nerve sheath meningiomas (ONSMs) are rare and benign tumors that affect the optic nerve. Although surgical decompression may be used for large tumors that cause mass effect on the surrounding structures, the mainstay of treatment is radiotherapy. We report the case of a 54-year-old female patient who presented with progressive vision loss due to a recurrent right ONSM despite fractionated radiotherapy eight years prior and the subsequent interval regression of the tumor. The optical coherence tomography at the time of recurrence revealed thinning of the right retinal nerve fiber layer. She underwent salvage stereotactic radiosurgery using a marginal dose of 15 Gy. At six months post-radiosurgery, the patient had a dramatic improvement in visual acuity and visual fields despite persistent thinning of the retinal nerve fiber layer. This case illustrates how salvage radiosurgery can be a useful treatment modality in these challenging situations. This tumor’s exophytic growth and the steep dose fall-off of Gamma Knife radiosurgery might favorably affect visual recovery. However, the outcomes of single-session radiosurgery for ONSMs should be further evaluated.

## Introduction

Primary optic nerve sheath meningiomas (ONSMs) are rare benign tumors comprising approximately 2% of all orbital tumors and representing less than 1-2% of all meningiomas [1]. Primary ONSMs typically arise from the intraorbital or intracanalicular optic nerve sheath, whereas secondary ONSMs originate from outside the orbit [2]. Commonly, primary ONSMs present with painless, progressive loss of visual acuity or visual field, while the classic triad includes visual loss, optic nerve atrophy, and optociliary shunt vessels [3].

Radiographically, ONSM appears as a contrast-enhancing mass encasing the optic nerve which is visualized as a “tram-tracking” sign on axial post-gadolinium T1-weighted magnetic resonance imaging (MRI). Because these clinical and radiographic features are distinctive, surgical biopsies are currently considered unnecessary given the associated risk of visual morbidities [3]. Management options of primary ONSMs include observation, surgery, and radiotherapy or multimodal management. Among them, observation may only be warranted for patients who have maintained the functional vision and agree with close clinical and radiographic surveillance [3]. Total resection is curative but associated with visual deterioration and thus only indicated in cases with poor vision at presentation [2,3]. Therefore, radiotherapy has been a mainstay of the treatment, providing favorable tumor control and possibly resulting in improvement of visual function or its stabilization [4,5]. Regarding the modalities of radiotherapy, several methods have been proposed including conventional fractionated radiotherapy via three-dimensional conformal radiotherapy or intensity-modulated radiation therapy (IMRT) and fractionated stereotactic radiotherapy (fSRT); however, little is known about outcomes of single-session radiosurgery [4]. Multiple studies have examined the pooled analysis of three-dimensional conformal radiotherapy, IMRT, fSRT, and radiosurgery showing that stereotactic radiotherapy or fractionated radiosurgery typically have the best long-term outcomes with 100% tumor control in small cohorts at a median of 68 months of follow-up [6-9].

Although radiotherapy for ONSMs generally provides good tumor control, tumors may recur, and treatment strategies for recurrent ONSMs have not been well established. Here, we present the case of a patient presenting with decreasing visual function and radiographic progression of a tumor toward the chiasm five years after IMRT using 50.4 Gy in 28 daily fractions. Since salvage with single-fraction stereotactic radiosurgery (SRS), she has maintained tumor control and has improved visual function.

## Case presentation

History and examination

A 54-year-old woman presented with subjective blurry vision and decreased visual acuity (20/150 OD) on visual examination eight years prior and was diagnosed with a primary ONSM. She underwent fractionated IMRT receiving 50.4 Gy in 28 fractions. Her vision improved (20/20 OD) post-therapy and she returned to her normal daily activities. Follow-up imaging demonstrated a decrease in tumor volume on MRI five years later. However, two years later, seven years after completing radiotherapy, the patient began to note further right-sided visual loss, and imaging demonstrated progression of the tumor consistent with late radiation failure. It was at this point she presented to our clinic. She was found to have a progressive ONSM with severe right-sided visual loss with hand motion vision only.

Investigation

**Figure 1:**
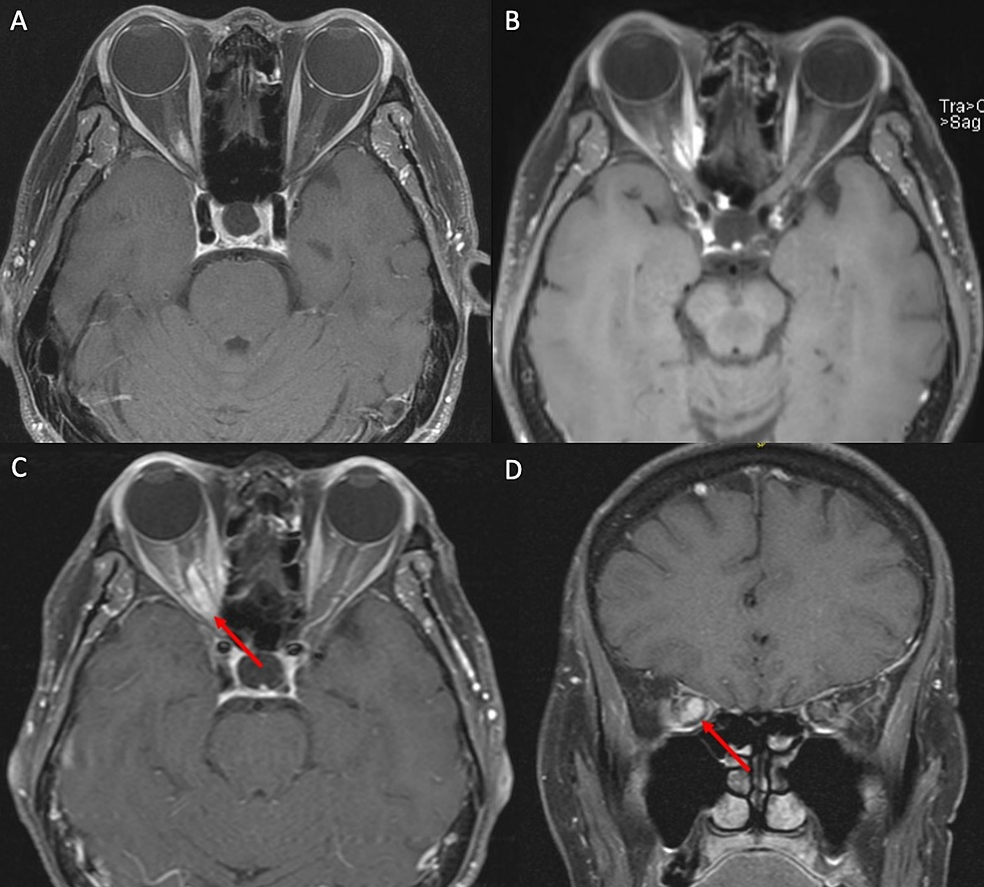
MRI demonstrating ONSM before and after fractionated radiotherapy. MRI demonstrated the tumor along the right optic nerve. (A) Initial axial T1 contrast-enhanced MRI upon presentation before radiotherapy demonstrated an enhancing lesion along the medial aspect of the optic nerve along the medial aspect of the orbital apex. (B) Five years after radiotherapy, the patient was noted to have stability on surveillance MRI without changes in vision. (C and D) Seven years after the initial presentation, the patient had 12 months of progressive vision loss with a more circumferential lesion (red arrow) prior to radiosurgery. MRI: magnetic resonance imaging; ONSM: optic nerve sheath meningioma

**Figure 2:**
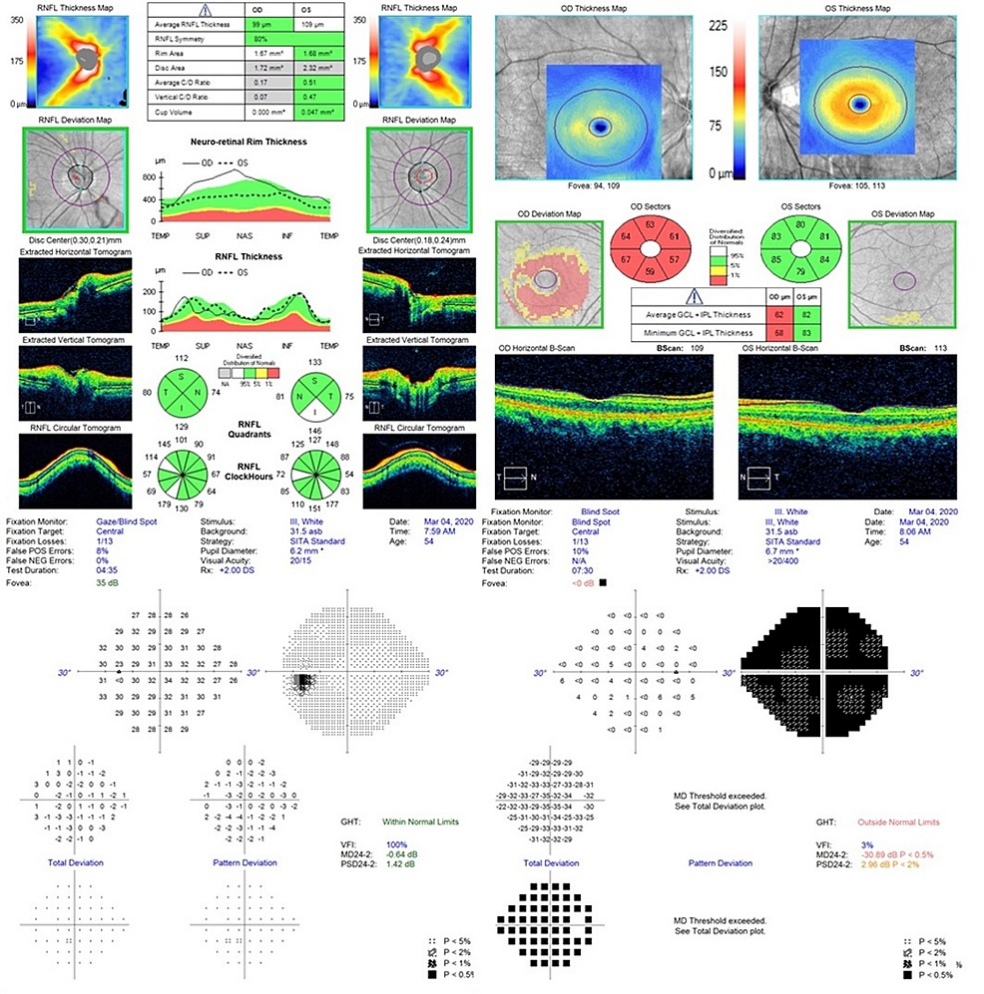
OCT and visual fields on presentation. OCT upon presentation to our clinic. The patient had undergone fractioned radiotherapy eight years prior. OCT demonstrated thinning of the right retinal nerve fiber layer at 99 µm, with thinning of the right ganglion cell layer and inner plexiform layer. Formal 24-2 Humphrey visual field assessment demonstrated a full visual field in the left eye, but diffusely decreased sensitivity in the right eye. OCT: optical coherence tomography

**Figure 3:**
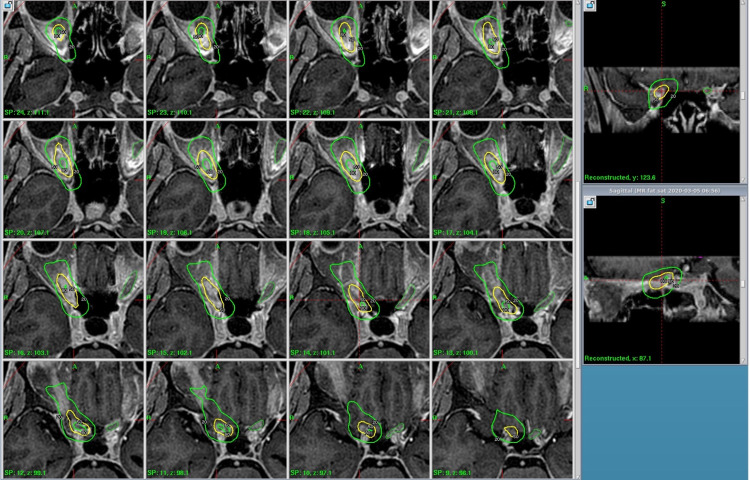
GKRS treatment plan. Axial T1 contrast-enhanced MRI with GKRS plan showing the 80% isodose, 50% isodose, and 20% isodose lines with the plan along and adjacent to the optic nerve. The prescription dose remained at 15 Gy to the 50% isodose line. GKRS: Gamma Knife radiosurgery; MRI: magnetic resonance imaging

A repeat brain MRI revealed a 17 mm linear enhancing lesion along the medial aspect of the intraconal right optic nerve extending back to the optic foramen. The tumor measured 4.5 × 6.5 × 17 mm, which was a progression from the five-year post-treatment image demonstrating a tumor that had shrunk to 2.5 × 5 × 10 mm from its original measurement of 3 × 6 × 10 mm (Figure 1). Visual acuity in the right eye was hand motion. The optical coherence tomography (OCT) (Figure 2) revealed thinning of the right retinal nerve fiber and ganglion cell layers, and the 24-2 Humphrey visual field test (Figure 2) demonstrated generalized decreased sensitivity in the ipsilateral eye. Fundoscopic examination demonstrated nerve pallor and no afferent pupillary defect. The treatment options were discussed with the patient. She elected to undergo Gamma Knife radiosurgery (GKRS). The Dmax to the optic nerve from prior radiotherapy was 11.2 Gy. The meningioma was treated with a prescription dose of 15 Gy at the 50% isodose line using nine isocenters and covering a volume of 1,750 mm^3^ (Figure 3) with an optic nerve Dmax of 16.2 Gy, aiming for definitive tumor control given the possible radiation resistance of the recurrent meningioma.

Follow-up evaluation

**Figure 4:**
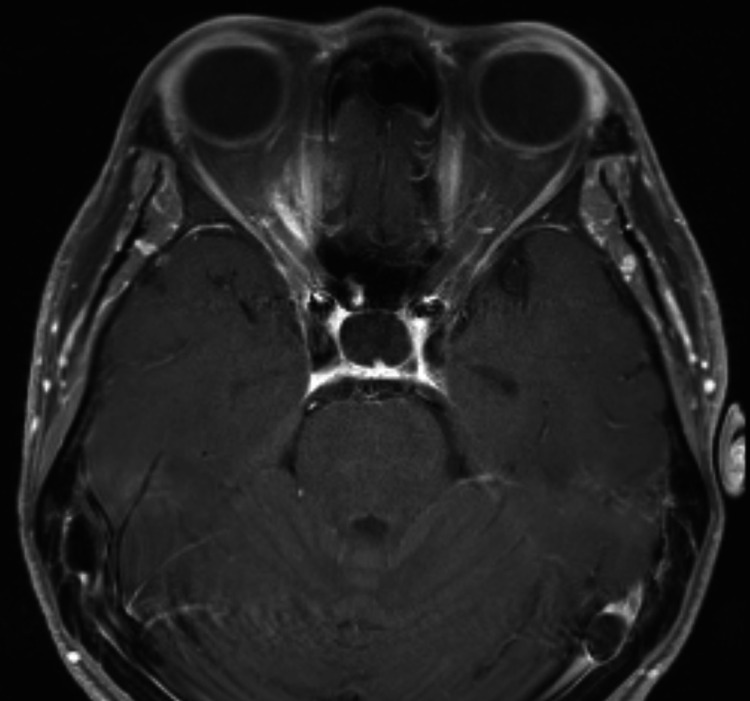
MRI nine months post-radiosurgery. Axial T1 contrast-enhanced MRI done nine months after GKRS demonstrating no significant change in the size of the tumor although it had a more diffuse appearance. GKRS: Gamma Knife radiosurgery; MRI: magnetic resonance imaging

**Figure 5:**
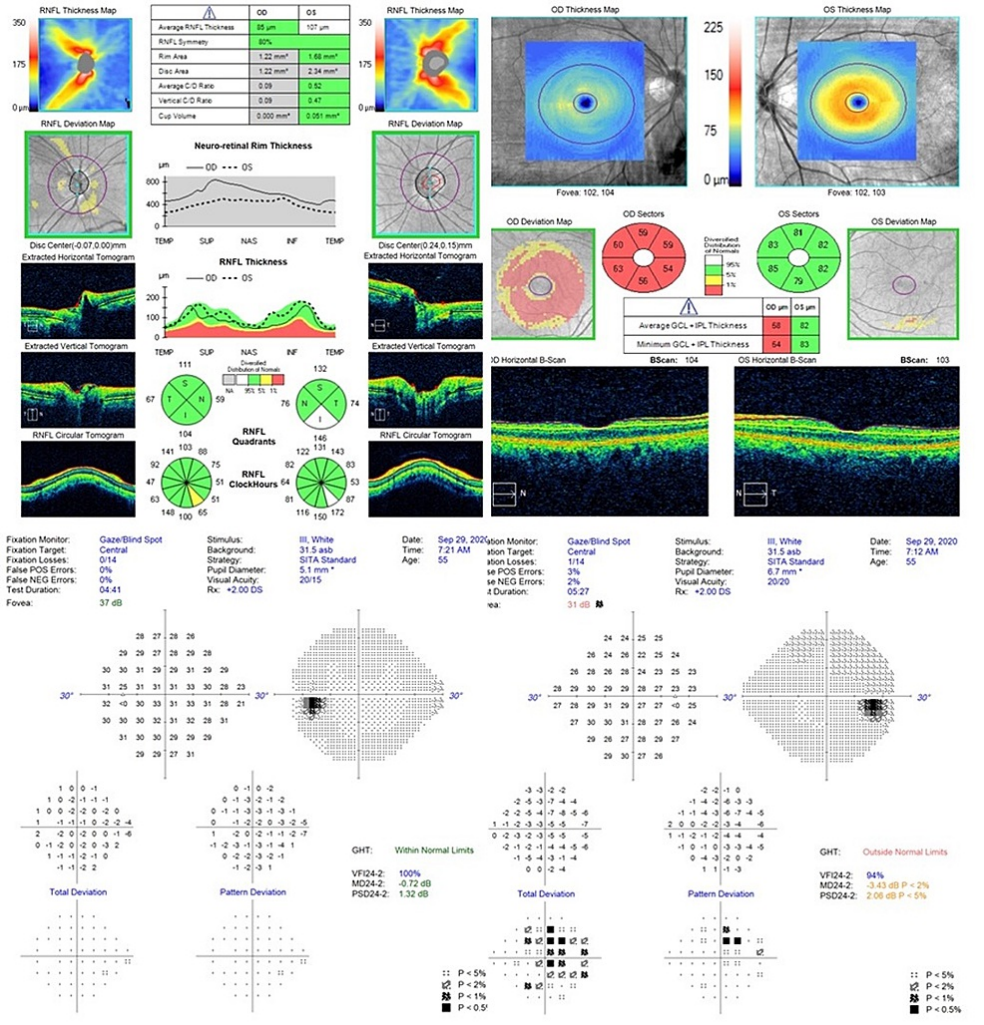
Post-radiosurgery OCT and visual fields. Post-radiosurgery OCT demonstrating a decrease in the right retinal nerve fiber layer to 85 µm with thinning of the right ganglion cell layer and inner plexiform layer. Formal Humphrey visual field tests performed at the six-month follow-up after GKRS demonstrating a normal visual field in the left eye and significantly improved sensitivity and visual field in the right eye. GKRS: Gamma Knife radiosurgery; OCT: optical coherence tomography

One week after GKRS, the patient started noticing an improvement in her depth perception and vision in her right eye. At six months after radiosurgery, the patient’s vision had significantly improved to 20/20^-1^ vision. MRI at the same time showed no significant change in either the size of the tumor, peritumoral edema, or changes suggestive of neurotoxicity to the optic nerve or optic apparatus. The tumor remained stable in overall size without any change in the mass effect upon the optic nerve (Figure 4). Although repeat OCT (Figure 5) showed persistent thinning of the retinal nerve fiber and ganglion cell layers, her visual field and visual acuity were markedly improved, with only trace depression of the visual fields (Figure 5). The patient continued to have improved vision without change at one year of overall follow-up duration after GKRS.

## Discussion

In this report, we treated a recurrent ONSM that caused profound vision loss using single-session GKRS which resulted in good tumor control and excellent vision recovery. The prescription dose of 15 Gy, which is a standard dose for benign meningiomas, was deemed necessary and reasonable for tumor control given the possible radiation resistance of the recurrent tumor following radiotherapy as well as the poor pretreatment visual testing and thinning of the retinal nerve fiber layer on OCT. The tumor remained well-controlled at one year, and the vision was fully recovered following GKRS despite repeat radiation exposure.

Radiation-induced optic neuropathy (RION) is the primary concern following radiotherapy for lesions close to the optic apparatus and is thought to be secondary to ischemia-related vascular injury resulting in optic nerve atrophy and injury to the neuronal elements [10,11]. In regard to radiation toxicity to the optic nerve in a single session, the existing literature suggests that 8-12 Gy to the optic nerve would be a borderline acceptable dose to avoid visual deficits [10,11]. Based on this, fractionated radiotherapy has been the standard of care for many lesions that are abutting or on the optic nerve because of the ability to deliver a higher dose over multiple fractions and reduce radiation injury to the nerve with the maximum dose being 12 Gy in one fraction, 20 Gy in three fractions, and 25 Gy in five fractions [10,12,13]. Indeed, there are numerous cases in the literature with ONSM demonstrating successful preservation of visual function following fractionated radiotherapy or hypofractionated SRT; however, visual outcome following single-session SRS remains unclear.

On the other hand, regarding tumor control, SRS has an excellent track record [14,15]. This theoretically makes sense because, in principle, meningiomas are benign tumors and are assumed to have a low alpha/beta ratio similar to normal benign tissues [15]. Previous studies suggest that a certain level of radiation dose, ranging from 12 to 18 Gy, is desirable to achieve favorable tumor control [14,15]. Moreover, radiation resistance in the tumor cells or alterations of the tumor microenvironment should also be considered given the tumor recurred following fractionated radiotherapy [16]. Therefore, we chose GKRS with the standard prescription dose of 15 Gy.

We do not have a clear explanation for the excellent visual outcome obtained in the present case. Although there is no good standard for pretreatment evaluation for prognosis of visual function [17-19], good pretreatment vision is reported to be a reasonable prognostic factor for good visual outcomes [8,9]. Moreover, a good appearance of the retinal nerve fiber layer on OCT is usually considered a sign of good potential recovery [18]. Thus, our case theoretically had a dismal pretreatment visual prognosis, which prompted a slightly more aggressive plan with a higher Dmax. Possible explanations for the good recovery are the high-precision treatment owing to GKRS and the tumor’s relatively exophytic growth pattern; however, this does not account for the high overall dose to the optic nerve. Due to the nature of the multi-isocenter treatment in gamma knife, the center of the target volume tends to be exposed to the highest radiation dose. Thus, when the tumor is circumferentially encasing the optic nerve, the nerve would receive radiation doses much higher than the doses most of the tumor receives. On the other hand, GKRS is characterized by the steepest dose fall-off across the radiotherapy modalities, and thus when the tumor shows exophytic growth away from the optic nerve, the adverse radiation effects could be minimized; however, this is just a case report and the outcomes of single-session radiosurgery for ONSMs should be further evaluated. Radiation-induced optic nerve toxicity may not be a simple issue as to the prescription dose or Dmax to the optic nerve being high or low.

Milano et al. reviewed 34 studies and 1,578 patients and found that prior resection in 76% of the patients did not correlate with any increased risk of RION but prior irradiation, seen in 6% of patients in this review, demonstrated a crude 10-fold increased risk of RION [20]. This in combination with the average dose to the optic apparatus in fractionated radiotherapy for ONSMs may warrant surgical intervention in select cases. The challenge with these complex decision algorithms also arises when taking into account prognostic factors such as poor pretreatment visual acuity and visual fields. In this minority of select patients, salvage radiosurgery may be warranted. In summary, this case is an important addition to the literature because it demonstrates a salvage therapy and the “gray” area in understanding and predicting visual outcomes from OCT.

## Conclusions

Managing previously radiated tumors poses a unique challenge. This case highlights some of the more challenging aspects of managing an ONSM that had previously undergone fractionated radiotherapy with good visual improvement and now underwent salvage SRS with a dramatic recovery of vision. Particularly for tumors that are adjacent to the optic apparatus or encompass it, as in this case, understanding and planning therapeutic options for patients who have already received a significant dose of radiation to the optic apparatus limits how aggressive one can be. Understanding some of the nuances of ONSMs and their presentation may allow for the use of salvage radiosurgery in cases that have been previously radiated with poor vision. In addition, this case highlights that OCT and visual fields alone may not always be predictive of RION, or the chance of recovery and salvage therapy should still be considered.

## References

[1] Soldà F, Wharram B, Gunapala R, Brada M (2012). Fractionated stereotactic conformal radiotherapy for optic nerve sheath meningiomas. Clin Oncol (R Coll Radiol).

[2] Shapey J, Sabin HI, Danesh-Meyer HV, Kaye AH (2013). Diagnosis and management of optic nerve sheath meningiomas. J Clin Neurosci.

[3] Eddleman CS, Liu JK (2007). Optic nerve sheath meningioma: current diagnosis and treatment. Neurosurg Focus.

[4] Liu D, Xu D, Zhang Z (2010). Long-term results of Gamma Knife surgery for optic nerve sheath meningioma. J Neurosurg.

[5] Liu JK, Forman S, Hershewe GL, Moorthy CR, Benzil DL (2002). Optic nerve sheath meningiomas: visual improvement after stereotactic radiotherapy. Neurosurgery.

[6] Ovens C, Dean B, Gzell C (2020). Optimal management in optic nerve sheath meningioma - a multicentre study and pooled data analysis. J Clin Neurosci.

[7] Parker RT, Ovens CA, Fraser CL, Samarawickrama C (2018). Optic nerve sheath meningiomas: prevalence, impact, and management strategies. Eye Brain.

[8] Pintea B, Boström A, Katsigiannis S, Gousias K, Pintea R, Baumert B, Boström J (2021). Prognostic factors for functional outcome of patients with optic nerve sheath meningiomas treated with stereotactic radiotherapy-evaluation of own and meta-analysis of published data. Cancers (Basel).

[9] Ratnayake G, Oh T, Mehta R (2019). Long-term treatment outcomes of patients with primary optic nerve sheath meningioma treated with stereotactic radiotherapy. J Clin Neurosci.

[10] Mohamed Ali A, Mathis T, Bensadoun RJ, Thariat J (2019). Radiation induced optic neuropathy: does treatment modality influence the risk?. Bull Cancer.

[11] Pollock BE, Link MJ, Leavitt JA, Stafford SL (2014). Dose-volume analysis of radiation-induced optic neuropathy after single-fraction stereotactic radiosurgery. Neurosurgery.

[12] Doroslovački P, Tamhankar MA, Liu GT, Shindler KS, Ying GS, Alonso-Basanta M (2018). Factors associated with occurrence of radiation-induced optic neuropathy at "safe" radiation dosage. Semin Ophthalmol.

[13] Ferguson I, Huecker J, Huang J, McClelland C, Van Stavern G (2017). Risk factors for radiation-induced optic neuropathy: a case-control study. Clin Exp Ophthalmol.

[14] Chung LK, Mathur I, Lagman C (2017). Stereotactic radiosurgery versus fractionated stereotactic radiotherapy in benign meningioma. J Clin Neurosci.

[15] Pikis S, Bunevicius A, Sheehan J (2021). Outcomes from treatment of asymptomatic skull base meningioma with stereotactic radiosurgery. Acta Neurochir (Wien).

[16] Aravindan N, Aravindan S, Pandian V, Khan FH, Ramraj SK, Natt P, Natarajan M (2014). Acquired tumor cell radiation resistance at the treatment site is mediated through radiation-orchestrated intercellular communication. Int J Radiat Oncol Biol Phys.

[17] Saeed P, Blank L, Selva D (2010). Primary radiotherapy in progressive optic nerve sheath meningiomas: a long-term follow-up study. Br J Ophthalmol.

[18] Park HH, Oh MC, Kim EH, Kim CY, Kim SH, Lee KS, Chang JH (2015). Use of optical coherence tomography to predict visual outcome in parachiasmal meningioma. J Neurosurg.

[19] Interlandi E, Pellegrini F, Papayannis A (2020). Optical coherence tomography angiography findings in optic nerve sheath meningioma. Case Rep Ophthalmol.

[20] Milano MT, Grimm J, Soltys SG (2021). Single- and multi-fraction stereotactic radiosurgery dose tolerances of the optic pathways. Int J Radiat Oncol Biol Phys.

